# Rethinking health-care systems to tackle social isolation and frailty

**DOI:** 10.1016/S2468-2667(25)00324-X

**Published:** 2026-02-06

**Authors:** Fereshteh Mehrabi, Mary Louise Pomeroy, Emiel O Hoogendijk, Thomas K M Cudjoe, Mark Oremus, Karen Bandeen-Roche, Charity Oga-Omenka

**Affiliations:** Department of Psychology, Concordia University, Montreal, QC, Canada (F Mehrabi PhD); Center for Equity in Aging, Johns Hopkins School of Nursing, Baltimore, MD, USA (M L Pomeroy PhD); Department of Epidemiology & Data Science, Amsterdam Public Health Research Institute, VU University Medical Center, Amsterdam UMC, Amsterdam, Netherlands (E O Hoogendijk PhD); Division of Geriatric Medicine and Gerontology, School of Medicine, Johns Hopkins University, Baltimore, MD, USA (T K M Cudjoe MD MPH); School of Public Health Sciences, University of Waterloo, Waterloo, ON, Canada (Prof M Oremus PhD, C Oga-Omenka PhD); Department of Biostatistics, Bloomberg School of Public Health, Johns Hopkins University, Baltimore, MD, USA (Prof K Bandeen-Roche PhD); Center on Aging and Health, Johns Hopkins University, Baltimore, MD, USA (T K M Cudjoe, Prof K Bandeen-Roche)

## Abstract

Ageing populations face increasing burdens from frailty and social isolation, which are two inter-related public health challenges that increase the risk of dementia, hospitalisation, and mortality. Despite health systems’ potential to intervene, the co-occurrence of frailty and social isolation remains overlooked in policy, research, and routine care, leading to fragmented and insufficient responses. Structural barriers (eg, cultural and linguistic obstacles, low health literacy, complex system navigation, financial constraints, geographical isolation, and care coordination) further limit access. In this Viewpoint, we highlight four priorities to address these challenges: (1) screening in primary and acute care; (2) integrated medical and social care; (3) social prescribing; and (4) equity-focused policy and research within ageing strategies. Coordinated and cross-sector action that addresses social determinants of health and embeds frailty prevention and social wellbeing as core health system functions is urgently needed to enable healthy ageing.

## Social isolation and frailty: intersecting public health challenges

Social isolation and frailty are distinct yet intertwined conditions that elevate the risk of dementia, hospitalisation, and mortality.^1–3^ Social health is now recognised as a key third pillar of health in addition to physical and mental wellbeing.^3,4^ Social isolation affects one in four adults aged 60 years and older,^5^ and frailty affects an estimated 12–24% of adults aged 60 years and older globally.^6^ Social isolation is associated with unhealthy behaviours such as poor nutrition, sedentary behaviour, and excessive alcohol consumption, which increase the risk of chronic diseases and accelerate physiological decline.^1,7,8^ Frailty—a state of reduced physiological reserve—further limits participation in social activities and exacerbates functional decline. Each condition is recognised as a public health priority.^1,3,9^

Using preliminary analyses of weighted 2023 National Health and Aging Trends Study data (unpublished), we estimated that approximately 14·7% of community-dwelling adults older than 65 years were both (pre)frail and socially isolated based on the frailty phenotype,^10^ and that 17% were both (pre)frail and socially isolated based on the frailty index.^11^ Evidence from the Longitudinal Aging Study Amsterdam suggests that frailty might precede and increase the risk of future social isolation over a 21year follow-up period, while social isolation worsens frailty in older age, creating a vicious cycle.^8^ Given that both social isolation and frailty develop gradually across the life course, their dynamic relationship^2,8^ underscores the importance of early detection and integrated approaches that promote healthy behaviours, maintain functional capacity, and address both conditions simultaneously to support healthy ageing.

These challenges highlight the potential role of health systems in mitigating the intersection of frailty and social isolation to prevent functional decline, reduce health-care costs, and support healthy ageing.^12^ Healthcare professionals are uniquely positioned to advance this effort due to their routine interactions with adults aged 65 years and older who are socially isolated and frail within health systems. Nevertheless, the capacity of health systems to respond to these challenges varies widely across countries, and the co-occurrence of social isolation and frailty, which is often overlooked in policy and research, might necessitate different strategies to meet a shared goal.

## The role of health care: missed opportunities in routine care

Despite the health sector’s strategic position, the identification and management of frailty and social isolation are not sufficiently integrated into routine care. Many older adults experiencing frailty and social isolation primarily interact with emergency departments or primary care providers, although people who are socially isolated might engage with routine care infrequently. Encounters in emergency and primary care settings offer crucial opportunities to reach people who are frail or socially isolated.^12^ Although interdisciplinary teams (eg, social workers, community health workers, and case managers) can extend beyond traditional settings to address physical and social determinants of health, these teams are inconsistently mobilised in healthcare systems.^12^ Tackling these challenges requires integrated, cross-sector approaches beyond traditional medicine^13^ that recognise the social roots of the links between frailty and social isolation.

## From hospital to home: rethinking recovery and social support systems

A key point arises when an older adult with frailty faces a stressor such as an injury, fall, hip fracture, or hospitalisation. Beyond immediate health risks, these events produce broader challenges, particularly the reliance on other people for recovery. Given diminished physiological reserves, older adults with frailty rely heavily on social support to compensate for physical vulnerabilities and aid rehabilitation.^14^ However, poor social networks can restrict the availability of social support and thereby delay recovery, worsen frailty, and increase rehospitalisation risk.^1^

Following hospital discharge, home and community-based services can play key roles in recovery and social reintegration. However, systemic barriers (eg, long waiting lists for medical and social services, workforce shortages, poor coordination, and unawareness of available services) often impede timely access to care.^15^ These challenges are especially acute for older adults with frailty who do not have access to informal caregivers or strong social support networks. In the USA, for example, adults older than 65 years without caregivers are often ineligible for skilled home health services.

## Structural barriers to health-care access

Barriers such as cultural and linguistic challenges, transportation difficulties in rural areas, unaffordable outofpocket costs, and poor health literacy limit access to care. Adults older than 65 years on lower incomes are also 10% more likely to experience social isolation.^1,16^ Adults older than 65 years might also struggle to identify available services or to navigate health systems that often overlook their unique circumstances.^16^

In publicly funded systems (eg, the UK and Canada), acute and hospital care are universally covered, but long-term care, which is crucial for managing frailty, often does not have sufficient and equitable funding. In the USA, although Medicaid covers home and community-based services, eligibility is limited to people on the lowest incomes.^17^ Such gaps create financial and logistical barriers that worsen health inequalities, increase hospital readmissions, and deepen social isolation and frailty.^1^

Overcoming these barriers would require that adults older than 65 years who are frail or isolated recognise their healthcare needs, navigate complex systems with inadequate support, overcome communication challenges, arrange transportation despite mobility issues, and manage costs.^1,18^ Closing these gaps is essential to enable care strategies that link medical care with social support. We propose four solutions: (1) screening, (2) integrated care, (3) social prescribing, and (4) policy and research.

## Screening as prevention

The interconnectedness between social isolation and (pre)frailty highlights the importance of detection, particularly because both social isolation and (pre)frailty are potentially reversible when identified early.^8,19^ Timely detection enables the deployment of holistic interventions that address physical, psychological, and social dimensions, ultimately improving wellbeing among older adults. Screening for social isolation and (pre)frailty, particularly in primary care settings, can facilitate proactive intervention and integration of medical, social, and community resources.

Frailty and social isolation often go undetected due to the absence of coordinated care pathways, absence of screening consensus, inconsistent documentation in medical records, focus on addressing specific clinical issues in visits with health professionals, and overlapping health concerns in older adults. Cognitive impairment and dementia, both modifiable risk factors for and potential outcomes of frailty and social isolation, further complicate early detection, especially when relying on selfreported measures.^1,16,18^

To improve detection, primary care and hospital settings should implement standardised screening protocols that assess core indicators of frailty and social isolation. Screening can be embedded into routine workflows and conducted by a multidisciplinary team, including nurses, physicians, or trained allied health professionals during intake, routine, and annual assessments, with social workers supporting followup and connection to community resources to ensure timely identification of people at risk of frailty and social isolation.^20^ Validated tools can assess cognitive impairment, mobility, and malnutrition, which are both risk factors for and outcomes of frailty.^18^ Measures such as gait speed provide a single indicator of physical performance, and the FRAIL scale^21^ and the Clinical Frailty Scale^22^ offer broader assessments based on functional status and clinical judgement. Although quick to administer, these tools require sufficient information on patients’ daily functioning, and their interpretation might vary across clinicians, highlighting the need for training and consistent application.^23^

No single tool is currently recommended for the routine clinical assessment of social isolation, as measures vary in focus, feasibility, and applicability to clinical care settings.^24^ Tools such as the CARED Social Isolation and Loneliness Referral Tool (assessing Connections, Activities, Relationships, Emergency contact, and Dwelling),^25^ the Lubben Social Network Scale,^26^ and the Berkman–Syme Social Network Index^27^ capture different structural and functional dimensions of social connection. The US National Academy of Medicine has recommended incorporating the Berkman–Syme Index into electronic health records to facilitate systematic screening and guide intervention planning.^28^ This approach has been implemented in large community health centre networks using EPIC (an electronic health record system widely used in US hospitals), providing a structured workflow to systematically screen patients for social isolation, review needs, identify referral options, place referrals, and track follow-up outcomes.^29^

Implementing frailty and social isolation assessments in busy clinical settings requires addressing practical and conceptual barriers, including time, financial constraints, and variability in tool administration. Workforce strategies, such as task-shifting to trained allied health staff, and consistent application throughout the care continuum (ie, at admission and amid discharge planning), can improve feasibility and ensure follow-up. These approaches help healthcare providers to identify social vulnerabilities, including financial hardship, limited healthcare access, caregiving responsibilities, and mistreatment, which facilitates timely and personcentred interventions. Effective implementation requires follow-through via comprehensive assessment, personalised care planning, health monitoring, and interdisciplinary coordination.^18^ Meanwhile, further research is needed to optimise screening, develop effective care plans, and determine which interventions are the most beneficial. Expanding frailty and social isolation protocols and improving access to home care and community-based care can alleviate healthcare burdens and enhance the quality of life for adults aged 65 years and older.

Several initiatives illustrate the feasibility of structured screening and integrated assessment for frailty and social isolation. The UK’s National Health System (NHS) Comprehensive Geriatric Assessment and the National Clinical Programme for Older People in Ireland exemplify multidisciplinary models that address frailty and its social dimensions through coordinated evaluation and followup care, improving outcomes and reducing hospitalisations.^30^ In the USA, the Centres for Medicare and Medicaid Services AgeFriendly Hospital Measure requires hospitals to implement protocols for screening frailty (including cognition and mobility) and social isolation.^18^ In Canada, the CARED referral criteria provide a practical approach to identifying people who are socially isolated and guiding referrals to community programmes when multiple criteria are met.^20^ Together, these initiatives show structured screening and integrated care approaches to capture medical and social determinants of frailty, promote inclusiveness, and improve health outcomes, high lighting how national policy can accelerate integrated screening.

## Integrated care across sectors

Integrated care models that combine clinical home care and social support show promise in addressing medical and social needs simultaneously.^1^ These approaches enable the early detection of social withdrawal and timely intervention before social isolation becomes severe. Recognising that adults aged 65 years and older vary widely in their abilities to articulate needs, navigate services, and engage with support, integrated care models need to be flexible and tailored to individual circumstances. A humancentred approach that leverages thoughtful design—a method that actively engages adults aged 65 years and older to identify needs and cocreate solutions—has the potential to encourage people and communities to fully participate in their care, make decisions that reflect their priorities, and engage with services in meaningful ways.^31^ Importantly, adults aged 65 years and older who are experiencing frailty and social isolation represent a diverse population with varying functional, social, and cultural needs that integrated care models need to accommodate. For example, the Program of AllInclusive Care for the Elderly^32^ in the USA integrates medical and social care to support adults aged 55 years and older with frailty to live independently. Brazil’s Family Health Strategy^33^ deploys community health workers to deliver homebased care and strengthen social support in underserved communities. In Canada, the Seniors’ Community Hub model links primary care teams with community and social services to deliver coordinated, holistic, and sustainable care for adults aged 65 years and older with frailty.^34,35^

Strengthening social support systems through evidencebased approaches (eg, social prescribing, caregiver support programmes, telehealth, and community-based physical and social activity interventions) can help to mitigate the physiological and psychological burdens of social isolation^36^ and frailty^37^ and ultimately foster better health trajectories and community reintegration. A coordinated response across medical, social, and community sectors ensures a holistic approach to the complex needs of adults aged 65 years and older who are frail and socially isolated. Such response also lays the foundation for more tailored strategies grounded in preventive care and social connectedness.

## Social prescribing

Social prescribing—which connects people with nonclinical resources such as exercise classes, cultural programmes, social groups, and volunteer opportunities—is a promising approach to address social determinants of health, and should be considered as a key component of integrated care. For adults aged 65 years and older who are socially isolated or (pre)frail, social prescribing can help to identify and address unmet social needs and fosters engagement and community ties. As a connection between clinical care and community supports, social prescribing has been linked to reduced hospital readmissions, lower healthcare utilisation, and improved quality of life.^38^ Evidence from programmes such as the Experience Corps trial shows that structured volunteer programmes can enhance cognitive health, reduce frailty and falls, improve mobility, and strengthen social engagement and psychological wellbeing among adults aged 60 years and older, underscoring the role of meaningful social participation in promoting healthy ageing.^39^

However, fragmentation between medical home care services and social support programmes hinders effective intervention, as these services often operate individually rather than within an integrated continuum. To address this issue, home care models need to incorporate social assessments, connectionorientated care planning, and formal community linkages. Global innovations show how social prescribing and integrated care can bridge medical and social sectors. In the UK, integrated care boards fund and coordinate networks of social prescribing link workers, who are embedded within Primary Care Networks as part of multidisciplinary teams. These roles are financed through NHS allocations for the national roll out of the link worker role and are supplemented by national contracts and data-sharing systems within integrated care systems. Workforce capacity is strengthened through a national competency framework that emphasises personcentred engagement and community development. However, variable outcome expectations, uneven data sharing, and regional differences in voluntarysector capacity limit the consistent impact of link workers across localities, highlighting the importance of aligning resources and information systems to reach consistent outcomes.^38,40^

In the Netherlands, the Buurtzorg model^41^ relies on decentralised, selfmanaged neigh bourhood nursing teams that are funded through governmentbacked insurance contracts and supported by streamlined digital tools that minimise administrative burden and enable holistic, communityorientated care. Despite strong outcomes, the Buurtzorg model operates within structural constraints, such as funding ceilings that limit scalability. Beyond the Netherlands, adaptation of this model is challenged by workforce shortages, cultural shifts required for self-management, the need for supportive governance, and IT infrastructure.^38,41^

Singapore’s Community Networks for Seniors rely on a robust community-based system that is centred on active ageing centres, coordinated by the Agency for Integrated Care and funded by the Ministry of Health. Workforce capacity is supported with structured training in digital literacy and leadership development. This model shows strong cross-agency integration and community engagement, yet its sustainability depends on continued government investment and effective cross-agency coordination, which might be vulnerable to policy or budgetary shifts, underscoring the need for stable funding and ongoing workforce development (table).^38^

Implementation of social prescribing remains uneven, particularly among populations that face structural barriers (eg, Indigenous communities, immigrants, people with disabilities, and people who are LGBTQIA+), who might experience heightened risks of frailty, social isolation, and poor access to health care.^1,3,4^ Addressing these barriers is key to strengthening preventive care and promoting health equity.

## Moving forward: policy, research, and system alignment to support healthy ageing

Sustained progress requires coordinated policy efforts and continued research investment to support the evidencebased, equitydriven integration of health and social care systems. The challenges of social isolation and frailty and structural inequities vary by context: high-income countries often face system fragmentation, and low-income and middleincome countries encounter constraints in infrastructure, financing, and access to health care. Strategies need to be tailored to local contexts and consider financing systems, healthcare infrastructure, and the needs of disproportionately affected populations (eg, Indigenous, rural, and some racial and ethnic communities) to ensure equitable and effective implementation. Integration principles emerging across various health and care frameworks—from singlepayer to multipayer models—show that coordinating medical and social care improves outcomes regardless of financing structure.^42^

To address these challenges, we propose a framework for evidencebased and equitydriven action. First, at the individual level, screening in primary and acute care can help identify adults aged 65 years and older who are at risk of social isolation and frailty, triggering timely, personcentred interventions. Second, at the health system level, integrated care connects healthcare services with supports through interdisciplinary teams, flexible care pathways, and approaches that accommodate diverse functional, social, and cultural needs. These connections can be coordinated by multidisciplinary teams who provide geriatric care, care coordinators, or designated liaisons, depending on local context (eg, multidisciplinary teams who provide geriatric care in high-income settings or task-shifting to trained community workers in low-income settings). Third, at the community level, social prescribing links people to community resources, including social groups, volunteer programmes, and physical activity initiatives (eg, formalised link workers in high-income settings or peerled groups in low-income settings). Fourth, at the population level, equity-focused policy and research—including rigorous evaluation and interventions—can embed social and functional health (defined as physical functioning and ability to perform daily actvities) into strategies for ageing populations and ensure that programmes are adaptable across diverse populations and health-care systems (eg, national frameworks with funding in high-income settings or integration into primary care platforms with donor support in low-income settings; figure).

Integrated care should adopt a personcentred approach that ensures interventions are accessible, equitable, and responsive to individual circumstances. Cross-sector collaboration, including health and social services, educational institutions, housing, transportation, community organisations, and the private sector, is needed to create a unified, societywide response.^3,4^ These strategies can help to tackle the cooccurrence of frailty and social isolation, promote healthy ageing, prevent hospitalisations, and support adults aged 65 years and older to remain functionally independent, socially connected, and engaged in their communities.

## Figures and Tables

**Figure:**
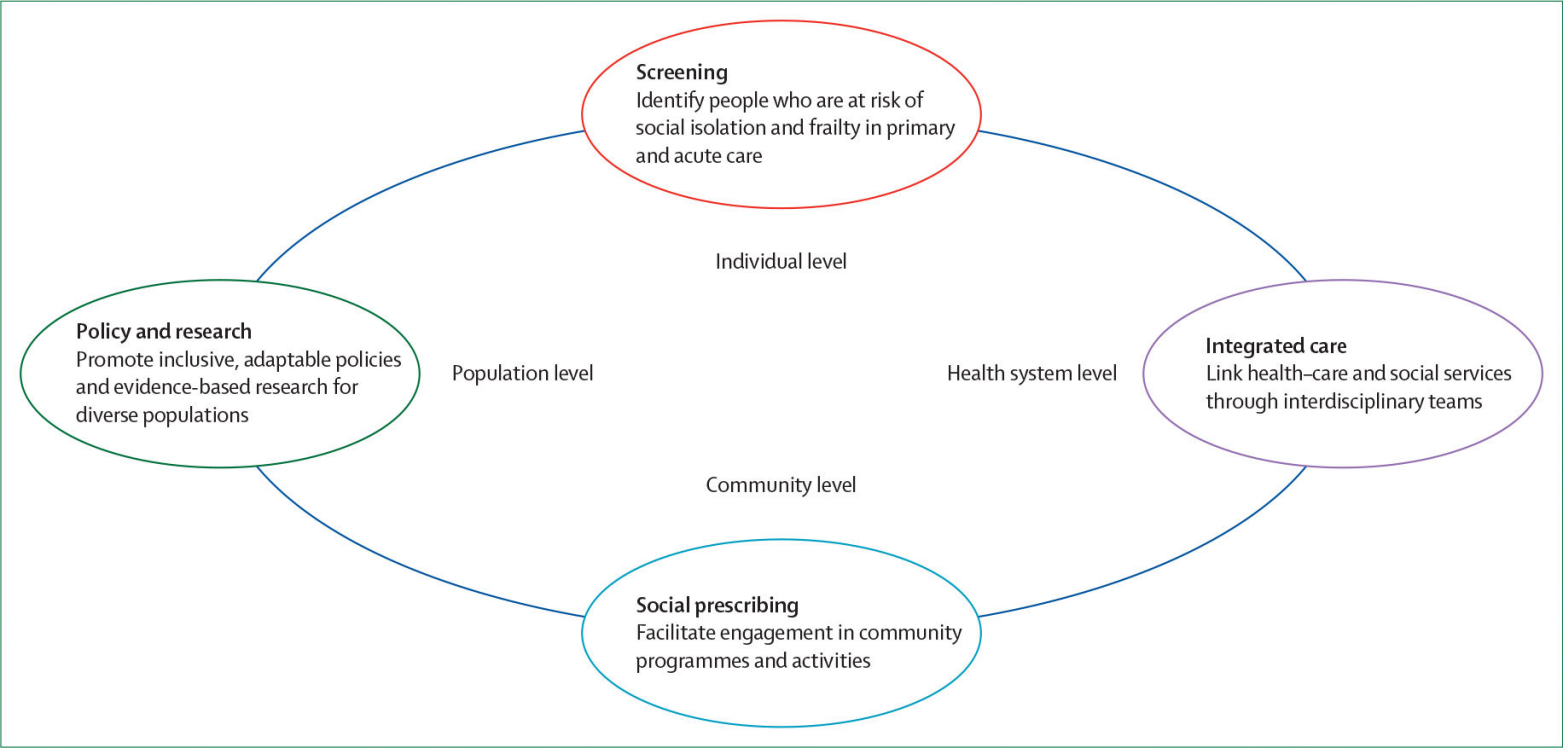
Framework to address social isolation and frailty

**Table:** Comparative summary of integrated care and social prescribing models

	Model	Key characteristics	Funding and infrastructure	Workforce competencies	Strengths	Limitations	Lessons for other systems
UK^40^	Social prescribing within integrated care boards	Link workers who are embedded in primary care	National funding; embedded link workers; data sharing via integrated care systems	National competency framework; personcentred practice	National scaling; formal integration with primary care	Variable voluntarysector capacity; uneven data-sharing	Align resources and data-sharing to ensure consistent outcomes across regions
Netherlands^41^	Buurtzorg neighbourhood nursing teams	Self-managed neighbourhood nursing teams	Insurance-based contracts	Skills in self-management, community nursing, and team decision making	Strong client outcomes; efficient staffing	Funding ceilings limit scalability	Transferability is limited by workforce and governance constraints
Singapore^NaN^	Community Networks for Seniors	Community-based network centred on active ageing centres coordinating health– social support	Ministry of Health funding; community hubs; coordinated by Agency for Integrated Care	Structured training in digital skills, leadership, and community engagement	Cross-agency integration; strong community infrastructure	Sustainability depends on continued government funding and coordination	Stable funding and cross-agency coordination are crucial for sustainability

## Search strategy and selection criteria

We searched PubMed and Google Scholar for articles, reviews, and commentaries published in English from database inception to Nov 25, 2025. We also manually reviewed reference lists of key publications and conducted targeted searches for governmental reports, policy briefs, and relevant webpages from WHO, national health agencies, and other international or professional organisations. Search terms included: (“social isolation” OR “social connection”) AND (“frailty” OR “older adults” OR “aging”) AND (“public health” OR “health policy” OR “healthcare systems”) AND (“social prescribing”) AND (“integrated care”) AND (“screening”). Articles and grey literature sources (including websites) were chosen based on their relevance to social isolation, frailty, and related interventions or policies.
